# Separation of Hydrogen from Carbon Dioxide through Porous Ceramics

**DOI:** 10.3390/ma9110930

**Published:** 2016-11-16

**Authors:** Taro Shimonosono, Hikari Imada, Hikaru Maeda, Yoshihiro Hirata

**Affiliations:** Department of Chemistry, Biotechnology, and Chemical Engineering, Kagoshima University, Kagoshima 890-0065, Japan; shimonosono@cen.kagoshima-u.ac.jp (T.S.); k2539294@kadai.jp (H.I.); k0099848@kadai.jp (H.M.)

**Keywords:** gas separation, hydrogen, carbon dioxide, porous ceramics

## Abstract

The gas permeability of α-alumina, yttria-stabilized zirconia (YSZ), and silicon carbide porous ceramics toward H_2_, CO_2_, and H_2_–CO_2_ mixtures were investigated at room temperature. The permeation of H_2_ and CO_2_ single gases occurred above a critical pressure gradient, which was smaller for H_2_ gas than for CO_2_ gas. When the Knudsen number (λ/*r* ratio, λ: molecular mean free path, *r*: pore radius) of a single gas was larger than unity, Knudsen flow became the dominant gas transportation process. The H_2_ fraction for the mixed gas of (20%–80%) H_2_–(80%–20%) CO_2_ through porous Al_2_O_3_, YSZ, and SiC approached unity with decreasing pressure gradient. The high fraction of H_2_ gas was closely related to the difference in the critical pressure gradient values of H_2_ and CO_2_ single gas, the inlet mixed gas composition, and the gas flow mechanism of the mixed gas. Moisture in the atmosphere adsorbed easily on the porous ceramics and affected the critical pressure gradient, leading to the increased selectivity of H_2_ gas.

## 1. Introduction

Currently, 90% of the hydrogen gas produced in the world is generated by a steam reforming process of naphtha (Equation (1)) followed by a water–gas shift reaction (Equation (2)):

(1) $$C_{m}H_{n}+mH_{2}O\to (m+n/2)H_{2}+mCO$$

(2) $$mCO+mH_{2}O\to mH_{2}+mCO_{2}.$$

The reformed gas contains hydrogen and carbon dioxide. The byproduct, carbon dioxide, is usually removed by a pressure swing adsorption method or a cryogenic distillation method at a low temperature. However, the separation of hydrogen from carbon dioxide at high temperatures of 200–400 °C (i.e., the reaction temperature of Equation (2)) using a membrane separator is desirable from the point of view of reducing the energy consumption and costs associated with hydrogen refinement.

Polyimide membranes are well-known commercialized membranes for separating hydrogen gas at operating temperatures less than 200 °C [1,2]. In polymer membranes used to separate gases, the gas molecules are transported through the spaces of the polymer chain network. Hydrogen molecules, which are small, permeate through the polymer membrane more easily than other gases. For example, when a mixture of 10 vol % H_2_ and 90 vol % CO_2_ was fed at 0.4–0.6 MPa to a polyimide membrane with a thickness of approximately 50 μm [1], the H_2_ selectivity (i.e., the ratio between the permeability coefficients of H_2_ and CO_2_) was 2.7, whereas the H_2_ permeability coefficient was approximately 3 × 10^−15^ mol·m/(m^2^·s·Pa) [1].

To enhance the H_2_ selectivity of polymer membranes, researchers have added metal [3] and have modified the surface of polymers with amine groups [4] to increase the membranes’ affinity toward CO_2_ gas and prevent the diffusion of CO_2_ molecules. At higher temperatures, a palladium metal membrane [5] or an amorphous silica membrane [6] is used, in which hydrogen atoms diffuse through atomic-sized spaces. In the Pd membrane, the dissociative dissolution of H_2_ molecules occurs selectively and is followed by the diffusion of H atoms. Studies of Pd membranes have focused on the fabrication of a thin Pd film without pinholes to increase the flux of permeated H_2_ gas and the H_2_ selectivity. Preventing delamination of the Pd membrane from its supporting material is important. Thus, researchers have developed Pd alloy membranes to suppress the phase transformation due to hydrogenation of Pd metal, which is accompanied by volume expansion [5,7]. The use of a metal support to reduce the mismatch between the thermal expansion coefficients of a Pd membrane and its supporting material has also been investigated [8]. In membranes with micropores (<2 nm), such as amorphous silica, smaller gas molecules move more easily through the pores. The challenge with such membranes is to prevent the collapse of the micropores at high temperatures (>450 °C); polysilazane-derived silicon carbonitrides have been reported to satisfy this requirement [9,10], and a further improvement in thermal stability of microporous structures by the addition of Ni has also been reported [11]. In these membranes, the selectivity of hydrogen gas is sufficiently high; however, a high flux of hydrogen gas is achieved only under a high pressure of a supplied gas.

A membrane with 10-nm-diameter pores is expected to exhibit a hydrogen gas flux greater than that in the aforementioned membranes. The flux (*J*) of gas through a porous material is expressed theoretically by the Knudsen or Poiseuille equation (Equation (3)):

(3) $$J=\alpha \frac{\Delta P}{L},$$

where α is the permeability coefficient of gas ($\alpha =2\varepsilon r\bar{c}/3RT$ for the Knudsen equation, $\alpha =\varepsilon r^{2}\bar{P}/8RT\eta$ for the Poiseuille equation); Δ*P*/*L* is the pressure gradient between the inlet and outlet gases; ε and *r* are the open porosity and pore radius of a porous membrane, respectively; $\bar{P}$ is the average pressure between the inlet and outlet gases; η is the viscosity of the gas; $\bar{c}$ is the average velocity of gas molecules ($\bar{c}=\sqrt{8RT/\pi M}$, where *M* is the molecular weight of the gas molecules); *R* is the gas constant; and *T* is the absolute temperature. The fraction (*F*) of hydrogen gas of the outlet gas for 50 vol % H_2_–50 vol % CO_2_ mixed gas is calculated by Equations (4) and (5) for Knudsen and Poiseuille flow, respectively:

(4) $$F_{K}(H_{2})=\frac{J(H_{2})}{J(H_{2})+J(CO_{2})}=\frac{\bar{c}(H_{2})}{\bar{c}(H_{2})+\bar{c}(CO_{2})}=\frac{\sqrt{M(CO_{2})}}{\sqrt{M(CO_{2})}+\sqrt{M(H_{2})}}$$

(5) $$F_{P}(H_{2})=\frac{J(H_{2})}{J(H_{2})+J(CO_{2})}=\frac{\eta (CO_{2})}{\eta (CO_{2})+\eta (H_{2})}.$$

The *F* value based on viscosities [12] of 8.73 μPa·s for H_2_ gas and 14.53 μPa·s for CO_2_ gas at 17 °C is 0.824 in Equation (4) and 0.611 in Equation (5). Note that the *F* value is related to the generally defined selectivity (i.e., the ratio between the permeability coefficients of the major and minor permeant gases) by Equation (6):

(6) $$f(H_{2})=\frac{\alpha (H_{2})}{\alpha (CO_{2})}=\frac{F(H_{2})}{1-F(H_{2})}.$$

**Figure 1:**
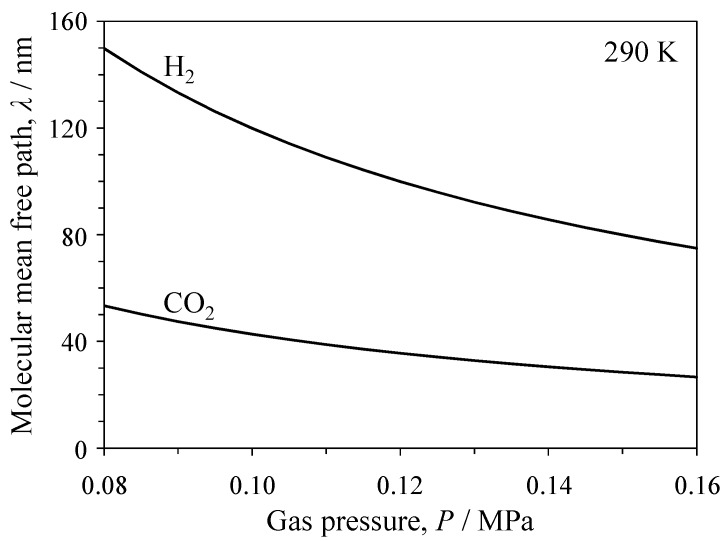
Molecular mean free paths of H_2_ and CO_2_ molecules as a function of gas pressure.

The ratio of molecular mean free path to the pore size (i.e., the Knudsen number) determines the mechanism of gas flow. Figure 1 shows the molecular mean free paths (λ) of H_2_ and CO_2_ gases calculated by Equation (7) at 290 K [13].

(7) $$\lambda =\frac{\eta }{0.499P\sqrt{8M/\left(\pi RT\right)}}$$

The λ value increases with decreasing gas pressure and is larger for H_2_ gas than for CO_2_ gas. When the λ/*r* (pore radius) ratio is less than unity, Poiseuille flow becomes dominant. For instance, H_2_ and CO_2_ gases exhibit Knudsen and Poiseuille flow, respectively, in a pore with an 80-nm radius (Figure 1), thereby influencing the flux of transported gas.

**Figure 2:**
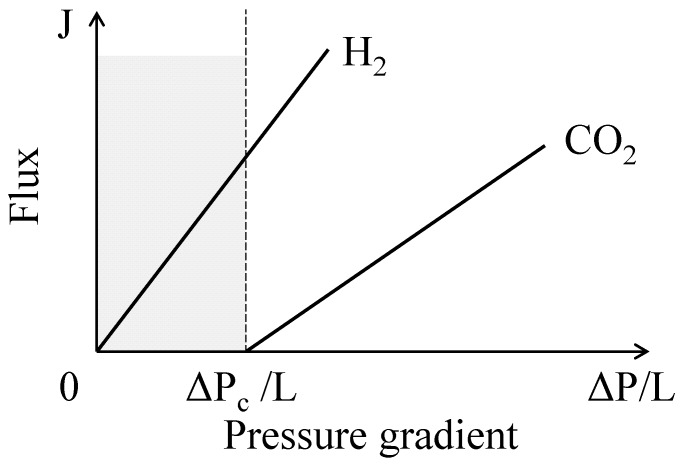
Schematic showing the flux–pressure gradient relation of H_2_ and CO_2_ gases through porous ceramics. A critical pressure gradient (∆*P*_c_/*L*) is observed for CO_2_ gas.

One approach to enhancing the selectivity of a gas permeated through porous ceramics is to modify the surfaces of ceramic particles by an agent that interacts with a gas and suppresses the permeation of the other mixed gas (e.g., AlOOH [14] and silane coupling agents [15] interact with CO_2_ gas). In this work, we used another approach to enhance the selectivity of a gas permeated through porous ceramics. In our previous study [16,17], the *J*–Δ*P*/*L* relation for CO_2_ gas was observed at pressures greater than a critical pressure gradient (Δ*P*_c_/*L*), as shown in Figure 2. However, the critical pressure gradient of H_2_ gas was closer to 0 MPa/m. This difference in critical pressure gradients can be exploited to separate H_2_ gas from CO_2_ gas in the Δ*P*/*L* range below the Δ*P*_c_/*L* for CO_2_ gas. A large difference in the Δ*P*_c_/*L* values for two gases also favors an increase in the flux of a selected gas at a relatively low pressure. In this study, the selectivity of the H_2_–CO_2_ mixed gas was investigated at room temperature using porous ceramics of α-alumina, yttria-stabilized zirconia (YSZ), and silicon carbide.

## 2. Experimental

### 2.1. Fabrication of Porous Ceramics

The employed powders were α-alumina (AKP50, specific surface area (*S*) 10.5 m^2^/g, equivalent diameter (*D*) 143 nm, isoelectric point (*iep*) pH 8.5, Sumitomo Chemical Co., Ltd., Tokyo, Japan), YSZ (TZ-8Y, 92 mol % ZrO_2_–8 mol % Y_2_O_3_, *S* = 14.9 m^2^/g, *D* = 68.3 nm, *iep* = pH 7.8, Tosoh Co., Tokyo, Japan) and SiC (impurity, mass%: SiO_2_ 0.66, C 0.37, Al 0.004 and Fe 0.013, *S* = 13.4 m^2^/g, *D* = 139 nm, *iep* = pH 2.5, Yakushima Electric Industry Co., Ltd., Tokyo, Japan). Each powder of Al_2_O_3_ and YSZ was dispersed at a solid content of 30 vol % in double-distilled water adjusted with an HCl solution at pH 3 where the particles were charged positively to increase the electrostatic repulsive energy among the particles. After stirring for 24 h, the suspensions were consolidated by pressure filtration at pressures as high as 19 MPa and were dried at 100 °C in air for 24 h. The obtained Al_2_O_3_ and YSZ compacts were sintered at 800 and 1100 °C, respectively, in air for 1 h. In the fabrication of porous SiC compacts, the sintering additives of α-alumina (AKP50) and yttria (purity > 99.9 mass%, *S* = 15.0 m^2^/g, *D* = 79.5 nm, *iep* = pH 7.5, Shin-Etsu Chemical Co., Ltd., Tokyo, Japan) were mixed in a mass ratio of SiC:Al_2_O_3_:Y_2_O_3_ = 1:0.02:0.02. The aqueous suspension was prepared at a solid content of 30 vol % at pH 5, where the positively charged sintering additives were attracted to the negatively charged SiC particles to form heterocoagulated particle clusters. After stirring for 24 h, the suspension was consolidated over a gypsum board. The obtained compacts were hot-pressed at 1400, 1500, or 1700 °C under a pressure of 39 MPa in an argon atmosphere for 2 h. The densities of fabricated compacts were measured by the Archimedes method using double-distilled water. The microstructures were observed by field-emission scanning electron microscopy (FE-SEM, S-4100H, Hitachi High-Technologies Co., Tokyo, Japan); the pore size distributions were analyzed via an intercept method [18]. The two-dimensional pore size determined by the intercept method was converted into a three-dimensional pore size by multiplying by a factor of 1.56, where the pores were assumed to be spherical [19]. The pore size distributions were also measured by mercury porosimetry at the Saga Ceramics Research Laboratory in Japan (Arita-cho, Nishimatsuura-gun, Saga 844-0024, Saga, Japan).

### 2.2. Gas Permeation in Porous Ceramics

**Figure 3:**
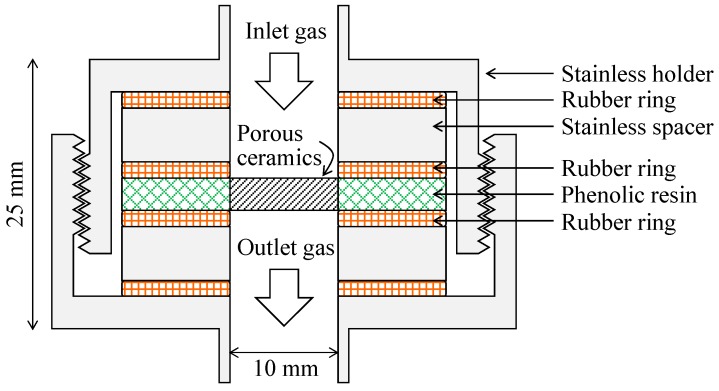
Gas permeation apparatus with porous ceramics.

The Al_2_O_3_ and YSZ samples were formed into disk shapes 20 mm in diameter and 3 mm in thickness. The diameter and thickness of the SiC disk sample were 10 and 3 mm, respectively. The side of each sample was sealed with phenolic resin. Before the gas separation experiments, each sample was dried at 100 °C for 24 h. The amount of moisture adsorbed on the dried sample was measured by a thermogravimetric/differential thermal analyzer (TG/DTA, Thermoplus, Rigaku, Tokyo, Japan). When the sample dried at 100 °C for 24 h was heated to 600 °C at the heating rate of 10 °C/min, the weight losses of Al_2_O_3_ and YSZ porous ceramics were about 0.25% and 0.20%, respectively. In the Al_2_O_3_ and YSZ samples dried at 300 °C for 24 h, the weight losses were about 0.25% and 0.10%, respectively. These results indicate that moisture in the atmosphere adsorbs easily on the present porous ceramics during the gas separation experiment. The sample to be measured was placed in a stainless steel holder, as shown in Figure 3. Rubber rings were placed in front of and behind the stainless spacers and were compressed by the stainless holder to avoid gas leakage. H_2_ (>99.999% purity), CO_2_ (>99.99% purity) and mixed gases with 20, 50, and 80 vol % H_2_ were supplied at pressures as high as 0.199 MPa at room temperature. The pressure of the permeated gas was approximately constant at 0.101 MPa. The gas pressure was monitored using a pressure gauge (MJE-PPX No. 0003-81V, CKD Co., Ltd., Aichi, Japan). The flux of permeated gas was measured using a flow meter (Soapfilm Flow Meter, 3001-11002, GL Science Inc., Tokyo, Japan). The compositions of inlet and outlet gases were measured using a gas chromatograph equipped with an active carbon column and a thermal conductivity detector (GT7100T, J-ScienceLab Co. Ltd., Tokyo, Japan). The temperatures of the column and detector were 70 and 100 °C, respectively. The electric current supplied to the detector was 60 mA, and the carrier gas was argon.

## 3. Results and Discussion

### 3.1. Microstructures of Porous Ceramics

**Figure 4:**
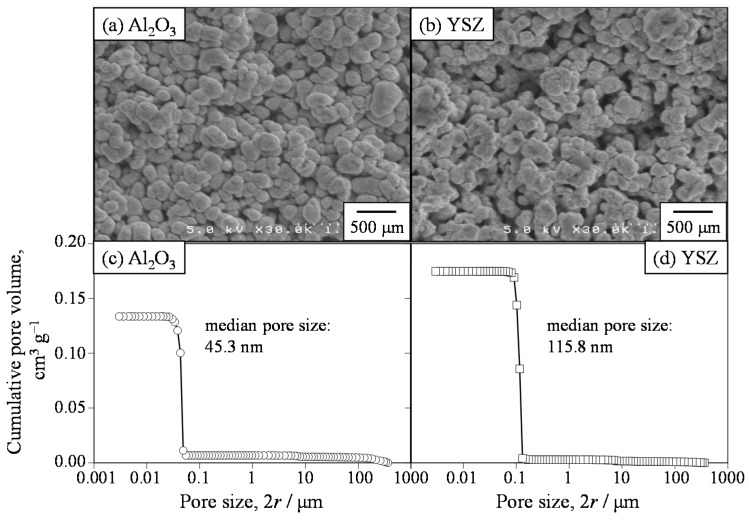
Microstructures (**a**,**b**) and pore size distributions (**c**,**d**) of Al_2_O_3_ and yttria-stabilized zirconia (YSZ) porous ceramics by mercury porosimetry.

Figure 4 shows the microstructures and pore size distributions of Al_2_O_3_ and YSZ compacts measured by mercury porosimetry. The relative density and the open and closed porosities were 62.7%, 36.2%, and 1.1%, respectively, for Al_2_O_3_ and 50.4%, 40.5%, and 9.1%, respectively, for YSZ. The median pore sizes of Al_2_O_3_ and YSZ were 45.3 and 115.8 nm, respectively. On the other hand, three-dimensional pore size was also determined on the observed microstructures (Figure 4a,b) by multiplying the two-dimensional pore size by 1.56, as previously discussed [16,19]. The pore sizes at 10% and 90% of the cumulative frequency were 10.8 and 133.9 nm, respectively, in porous Al_2_O_3_ and 8.2 and 113.1 nm, respectively, in porous YSZ. The median sizes of the pores of Al_2_O_3_ and YSZ were 49.4 and 39.1 nm, respectively. Compared to the pore size measured by microscopic technique, the pore size of mercury porosimetry was almost the same for the Al_2_O_3_ compact but was three times larger for the YSZ compact. In this paper, the pore size measured by mercury porosimetry was used to discuss the flux and permeability coefficient of the gas permeated through the porous ceramics.

**Figure 5:**
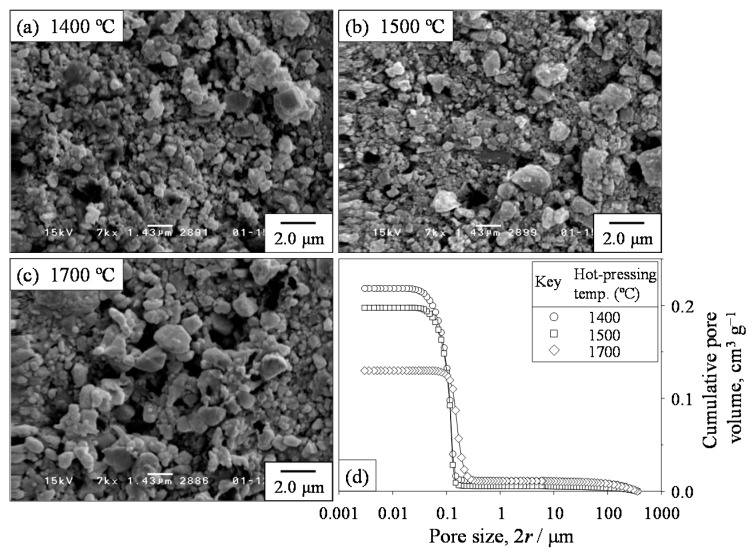
(**a**–**c**) Microstructures and (**d**) pore size distributions of SiC ceramics hot-pressed at (**a**) 1400 °C; (**b**) 1500 °C; and (**c**) 1700 °C.

Figure 5 shows the microstructures and pore size distributions of SiC samples hot-pressed at 1400, 1500, and 1700 °C. The relative density and the open and closed porosities of SiC samples were 61.1%, 36.1%, and 2.8%, respectively, at the hot-pressing temperature of 1400 °C, 69.3%, 28.1%, and 2.6%, respectively, at 1500 °C, and 75.4%, 17.9%, and 6.7%, respectively, at 1700 °C. The SiC samples densified through liquid-phase sintering consisted of bimodal particles smaller and larger than 1 μm. The fraction of larger particles increased at a higher hot-pressing temperature. Because the exact pore size distribution was difficult to measure on the basis of the scanning electron micrographs in Figure 5, the pore sizes were measured by mercury porosimetry. Figure 5d shows the cumulative pore volume of the porous SiC compacts. The median sizes of the cumulative pore volume were 111.9, 113.8, and 164.0 nm in the SiC samples hot-pressed at 1400, 1500, and 1700 °C, respectively. As a result, the pore size in the present experiment increased in the order Al_2_O_3_ < SiC ≈ YSZ.

### 3.2. Permeation of H_2_ and CO_2_ Gases through Porous Ceramics

**Figure 6:**
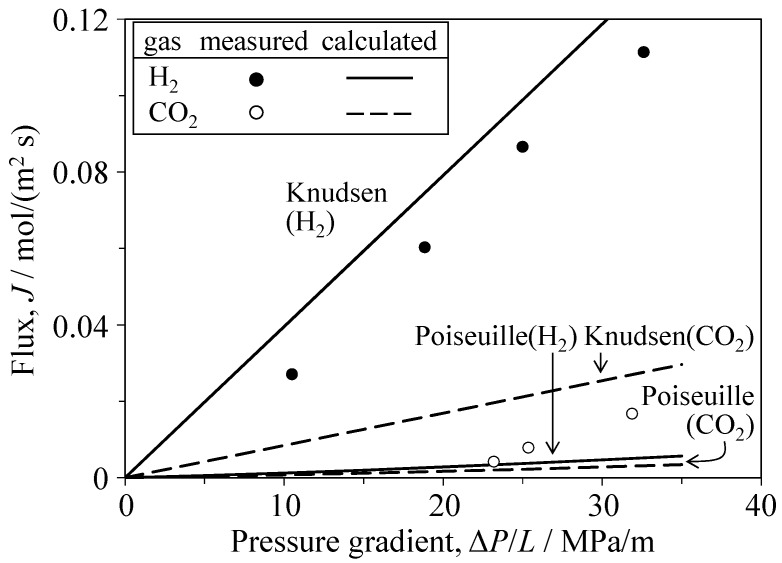
Single-gas flux of H_2_ and CO_2_ through Al_2_O_3_ porous ceramics as a function of the pressure gradient.

**Figure 7:**
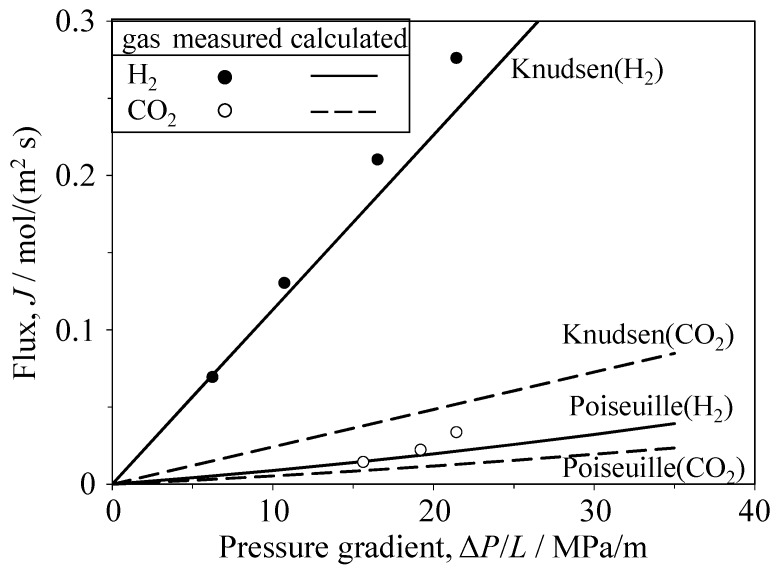
Single-gas flux of H_2_ and CO_2_ through YSZ porous ceramics as a function of the pressure gradient.

Figure 6 and Figure 7 show the single gas flux of H_2_ and CO_2_ through the Al_2_O_3_ and YSZ porous ceramics, respectively. The flux values for H_2_ and CO_2_ gases calculated via Equation (3) using the measured porosity and median pore size are also plotted in Figure 6 and Figure 7. The permeation of CO_2_ gas in the porous Al_2_O_3_ and YSZ occurred above a critical pressure gradient. The gas flux then increased linearly with increasing pressure gradient. Compared with the flux and critical pressure gradient measured for CO_2_ gas, those measured for H_2_ were larger and smaller, respectively. The critical pressure gradient values of H_2_ and CO_2_ gases were 3.2 and 20.0 MPa/m, respectively, for Al_2_O_3_ and 1.1 and 11.4 MPa/m, respectively, for YSZ. The measured flux of H_2_ gas through the porous Al_2_O_3_ was similar to the calculated flux based on Knudsen flow (Equation (3)). The measured flux of CO_2_ gas was within the values calculated for Knudsen flow and Poiseuille flow. The Knudsen number (=λ/*r*, Figure 1) was calculated to be 3.32–4.19 for H_2_ gas and 1.19–1.30 for CO_2_ gas; the exact values depend on the gas pressure (i.e., variation of the molecular mean free path in Figure 1), supporting the agreement between the experimental and the calculated transport characteristics of both gases. In the porous YSZ, the measured flux of H_2_ gas was close to the flux calculated using the Knudsen equation. Furthermore, the measured CO_2_ flux was within the values of CO_2_ flux calculated using the Knudsen and Poiseuille equations.

**Figure 8:**
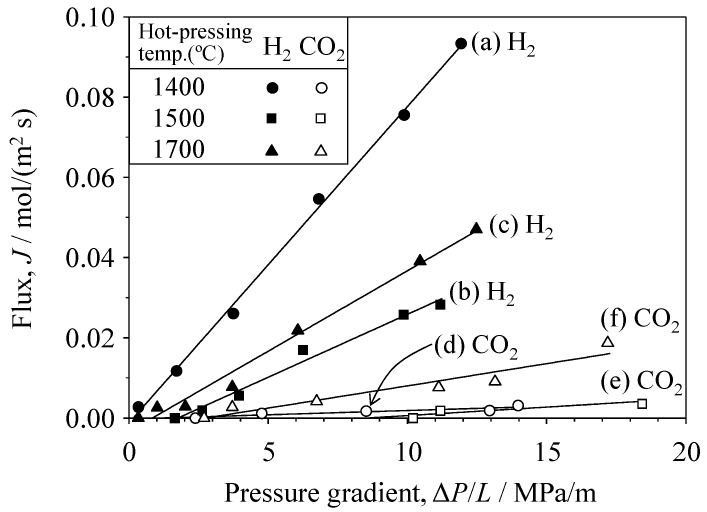
Single-gas flux of H_2_ and CO_2_ through porous SiC ceramics hot-pressed at 1400, 1500, and 1700 °C as a function of the pressure gradient.

**Figure 9:**
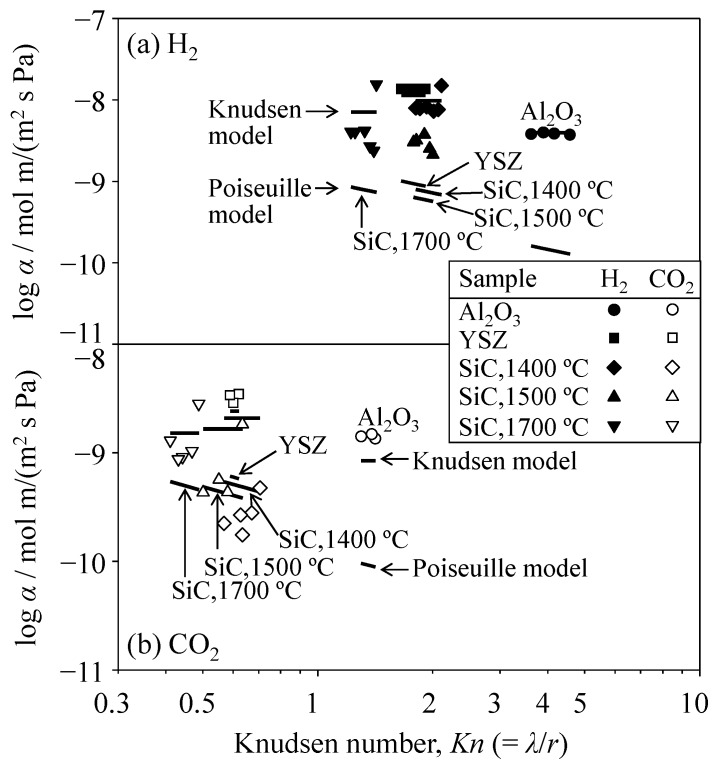
Permeability coefficients of (**a**) H_2_ gas and (**b**) CO_2_ gas in Al_2_O_3_, YSZ, and SiC porous ceramics against the pressure gradient above the Δ*P*_c_/*L* value as a function of the Knudsen number.

Similarly, Figure 8 shows the relationship between the single-gas flux of H_2_ and CO_2_ and the pressure gradient in the SiC porous ceramics. A similar trend of Δ*P*_c_/*L* (critical pressure gradient) for H_2_ and CO_2_ gases was observed in the porous SiC ceramics. The critical pressure gradient of H_2_ and CO_2_ gases was 0.2 and 2.4 MPa/m, respectively, in the SiC hot-pressed at 1400 °C, 1.7 and 8.5 MPa/m, respectively, at 1500 °C, and 0.8 and 2.8 MPa/m, respectively, at 1700 °C. Figure 9 and Table 1 summarize the permeability coefficients of H_2_ and CO_2_ gases as a function of the Knudsen number. The permeability coefficient was determined by dividing the flux *J* by the effective pressure gradient (Δ*P*/*L* − Δ*P*_c_/*L*). Note that, because each *J*–Δ*P*/*L* plot for H_2_ and CO_2_ gases showed an intercept (i.e., a critical pressure gradient), the permeability coefficient was determined for the gas flow above the Δ*P*_c_/*L* value. The Knudsen number represents the molecular mean free path (λ) normalized by the pore radius (*r*). The molecular mean free path was calculated using Equation (7). The pore radii of the oxides and SiC were measured by mercury porosimetry, as described in Section 3.1. The measured permeability coefficients were compared with the permeability coefficients calculated using the Knudsen and Poiseuille equations. In the Al_2_O_3_ and YSZ porous ceramics, the permeability coefficients of both the H_2_ and CO_2_ gases were similar to those calculated using the Knudsen model. In the SiC porous ceramics, the measured permeability coefficient of H_2_ gas is well explained by the Knudsen model. The permeability coefficient of CO_2_ gas was within the values predicted by the Knudsen and Poiseuille models. When the Knudsen number (λ/*r*) is greater than unity, Knudsen flow becomes the dominant gas transportation process, which is the usual tendency. As observed in Figure 9, when the Knudsen number decreases to a smaller value (<1) in the SiC porous compacts, the influence of Poiseuille flow becomes larger in the case of CO_2_ gas.

**Figure 10:**
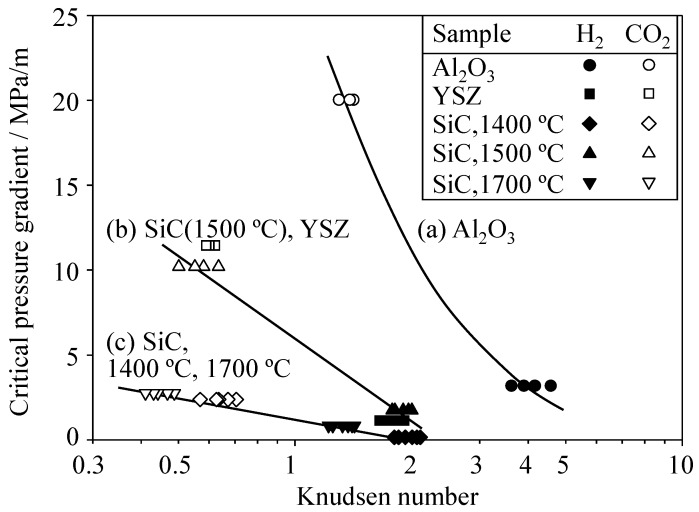
Relationship between the critical pressure gradient and the Knudsen number for (**a**) Al_2_O_3_, (**b**) YSZ and SiC sintered at 1500 °C, and (**c**) SiC sintered at 1400 °C and 1700 °C.

Figure 10 shows the critical pressure gradient (Δ*P*_c_/*L*) as a function of the Knudsen number (λ/*r* ratio). The Δ*P*_c_/*L* value increases at a small Knudsen number. This result implies that the Δ*P*_c_/*L* value is greater for CO_2_ gas with a small λ value than for H_2_ gas with a large λ value (Figure 1) in the same pore size distribution. Another observation is that the decreased pore size (2*r*) for a specified gas leads to an increase of the Δ*P*_c_/*L* value. That is, the Δ*P*_c_/*L* value increases in the case of Poiseuille flow giving a small flux of permeated gas rather than in the case of Knudsen flow giving a large flux of permeated gas (Figure 6 and Figure 7). As evident in Figure 10a, the Δ*P*_c_/*L* value is more sensitive to the Knudsen number in the case of the alumina porous ceramics than in the case of the SiC or YSZ porous ceramics. That is, the pore size, chemical composition of porous ceramics, and shape of pores (in SiC compacts) affect the Δ*P*_c_/*L* value. The large difference in Δ*P*_c_/*L* values between H_2_ and CO_2_ gases is effective to separate H_2_ gas from CO_2_ gas.

### 3.3. Permeation of H_2_–CO_2_ Mixed Gas in Porous Ceramics

**Figure 11:**
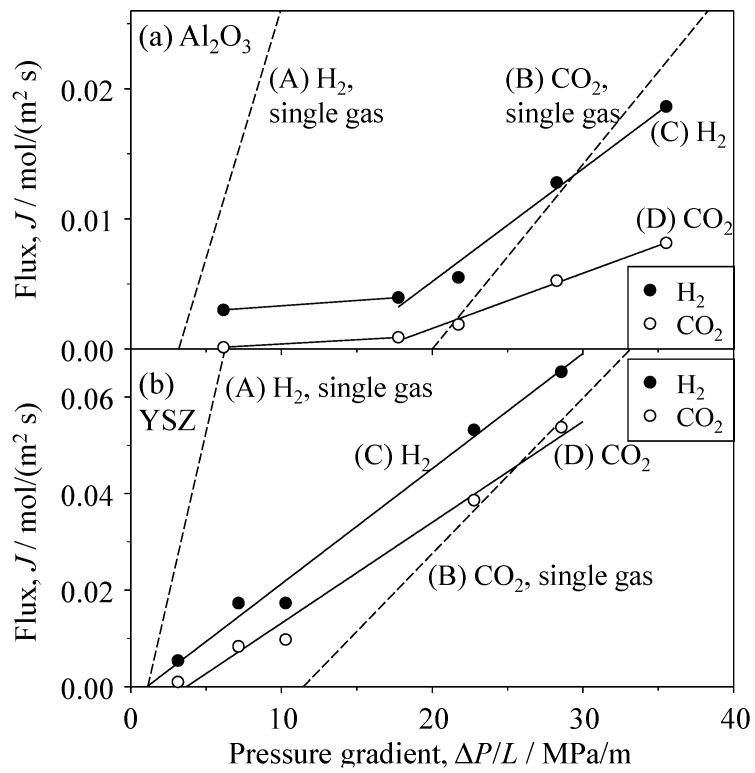
Flux of H_2_ and CO_2_ gases for the mixed gas of 50 vol % H_2_–50 vol % CO_2_ through (**a**) Al_2_O_3_ and (**b**) YSZ porous ceramics.

**Figure 12:**
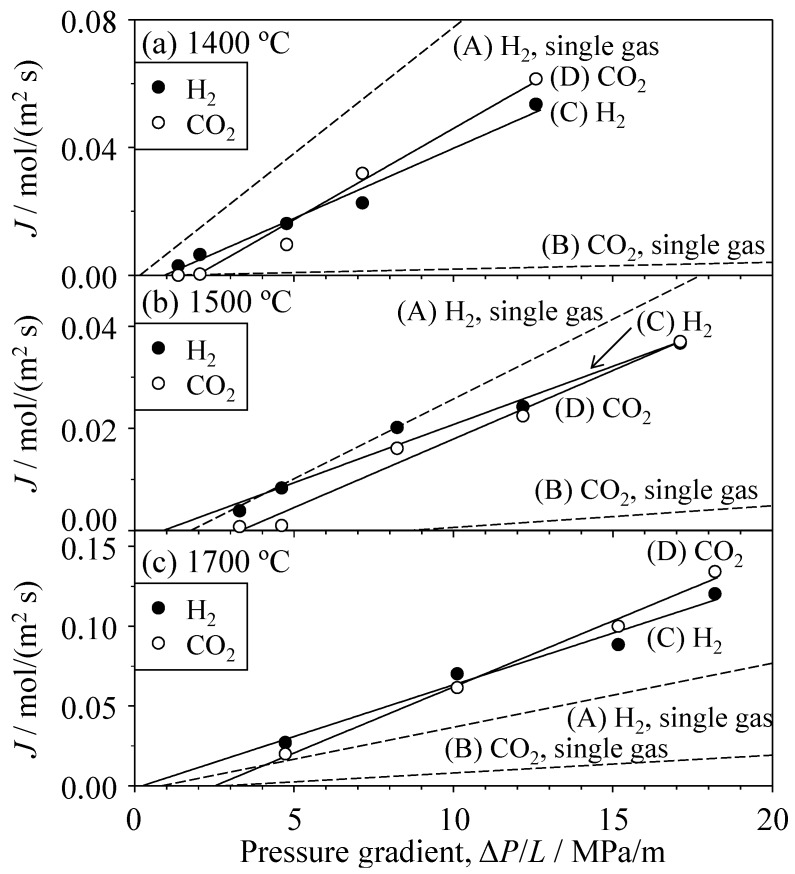
Flux of H_2_ and CO_2_ gases for the mixed gas of 50 vol % H_2_–50 vol % CO_2_ through SiC ceramics hot-pressed at (**a**) 1400 °C; (**b**) 1500 °C; and (**c**) 1700 °C.

Figure 11 shows the flux of H_2_ and CO_2_ gases for the mixed gas of 50 vol % H_2_ and 50 vol % CO_2_ in the Al_2_O_3_ and YSZ porous ceramics. For comparison, the flux values of H_2_ and CO_2_ single gases are also shown in Figure 11. In both of the porous ceramics, the flux of H_2_ gas was greater than that of CO_2_ gas. The flux of H_2_ gas in the mixed gas decreased substantially compared to that of single H_2_ gas. The critical pressure gradient (Δ*P*_c_/*L*) of CO_2_ gas in the mixed gas became smaller than that of single CO_2_ gas and approached that of single H_2_ gas. Figure 12 shows the flux of H_2_ and CO_2_ gases in the mixed gas permeated through the SiC porous ceramics. The flux values of H_2_ and CO_2_ gases were similar to each other and were similar in magnitude to the flux of single H_2_ gas. This tendency differs from the results shown in Figure 11. As previously mentioned, the H_2_ and CO_2_ single gases permeated through the Al_2_O_3_ or YSZ porous ceramics according to the Knudsen mechanism (Figure 9, Table 1). One factor used to interpret the permeation of the mixed gas is the interaction between pore walls and gas species, which is not expressed in the Knudsen equation (Equation (3)). The oxide porous ceramics have ionic bonds, and CO_2_ molecules are polarized between their carbon atom (delta plus) and oxygen atoms (delta minus) because of their different electronegativities. The attractive interaction between pore walls of porous oxide ceramics and CO_2_ molecules is expected to be strong compared to the interaction between pore walls and H_2_ molecules. The adsorption of CO_2_ molecules of the mixed gas on the pore walls of oxide ceramics may be interrupted by the collision between pore walls and H_2_ molecules, leading to a decrease in the critical pressure gradient of CO_2_ gas in the mixed gas.

**Figure 13:**
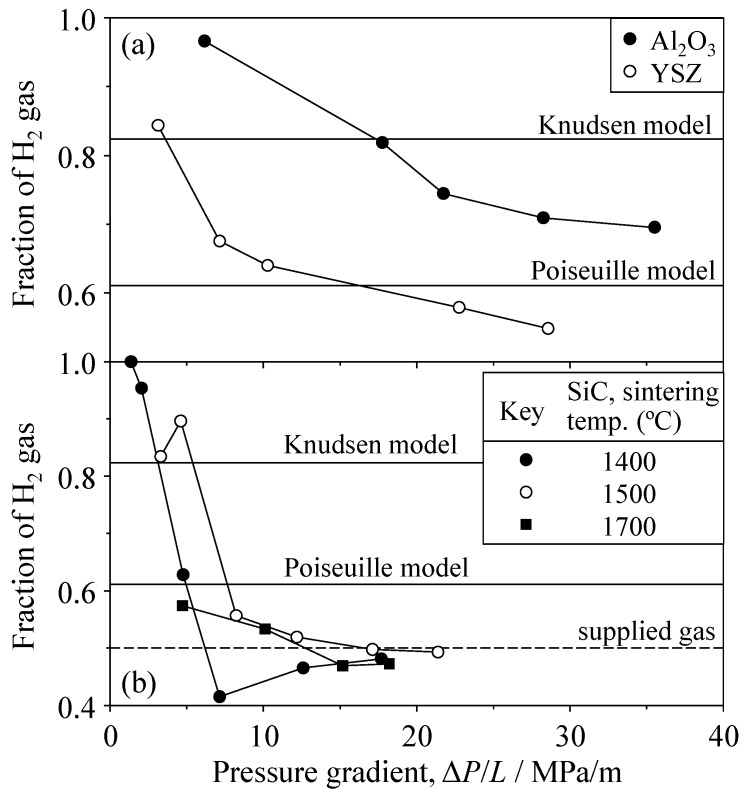
Fraction of H_2_ gas for the 50 vol % H_2_–50 vol % CO_2_ mixed gas through (**a**) Al_2_O_3_ and YSZ and (**b**) SiC.

On the basis of the data in Figure 11 and Figure 12, Figure 13 shows the fraction of H_2_ gas in the outlet gas and the H_2_ fractions predicted by the Knudsen and Poiseuille models. In the Al_2_O_3_ and YSZ porous ceramics, the H_2_ fraction was higher than that of the supplied gas. When the pressure gradient decreased, the H_2_ fraction increased, reaching 0.966 at 6.16 MPa/m in the porous Al_2_O_3_ and 0.844 at 3.13 MPa/m in the porous YSZ, which exceeded the fraction of H_2_ predicted by the Knudsen equation. In the SiC porous ceramics, the H_2_ fraction was approximately the same value as in the supplied gas at pressure gradients greater than 10 MPa/m. In the lower pressure-gradient range, the H_2_ fraction increased drastically. Within the detection limit of gas concentration (approximately 0.1% in this study), the H_2_ fraction reached almost unity at 1.36 MPa/m.

**Figure 14:**
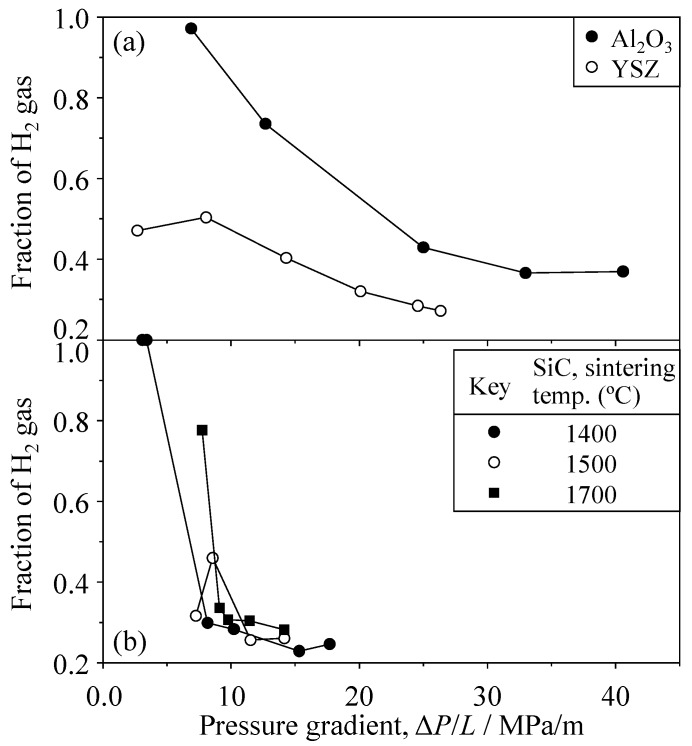
Fraction of H_2_ gas for the 20 vol % H_2_–80 vol % CO_2_ mixed gas through (**a**) Al_2_O_3_ and YSZ and (**b**) SiC.

**Figure 15:**
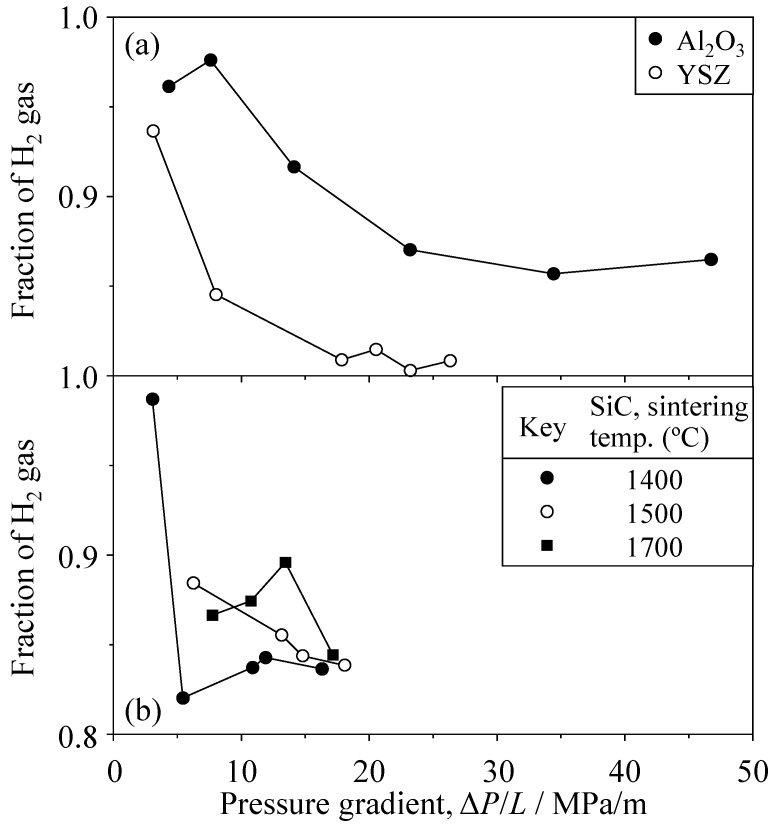
Fraction of H_2_ gas for the 80 vol % H_2_–20 vol % CO_2_ mixed gas through (**a**) Al_2_O_3_ and YSZ and (**b**) SiC.

Figure 14 and Figure 15 show the H_2_ fraction for 20 vol % H_2_–80 vol % CO_2_ mixed gas and 80 vol % H_2_–20 vol % CO_2_ mixed gas, respectively. A similar increase of H_2_ fraction was measured at a low pressure gradient. In the case of 20% H_2_–80% CO_2_ mixed gas (Figure 14), the H_2_ fraction was 0.972 at 6.88 MPa/m in Al_2_O_3_, 0.504 at 8.04 MPa/m in YSZ, and 0.999 at 3.40 MPa/m in SiC, respectively. When 80% H_2_–20% CO_2_ mixed gas was permeated (Figure 15), the H_2_ fraction was 0.976 at 7.61 MPa/m in Al_2_O_3_, 0.936 at 3.13 MPa/m in YSZ, and 0.987 at 3.06 MPa/m in SiC, respectively. These results are further discussed in a later section.

**Figure 16:**
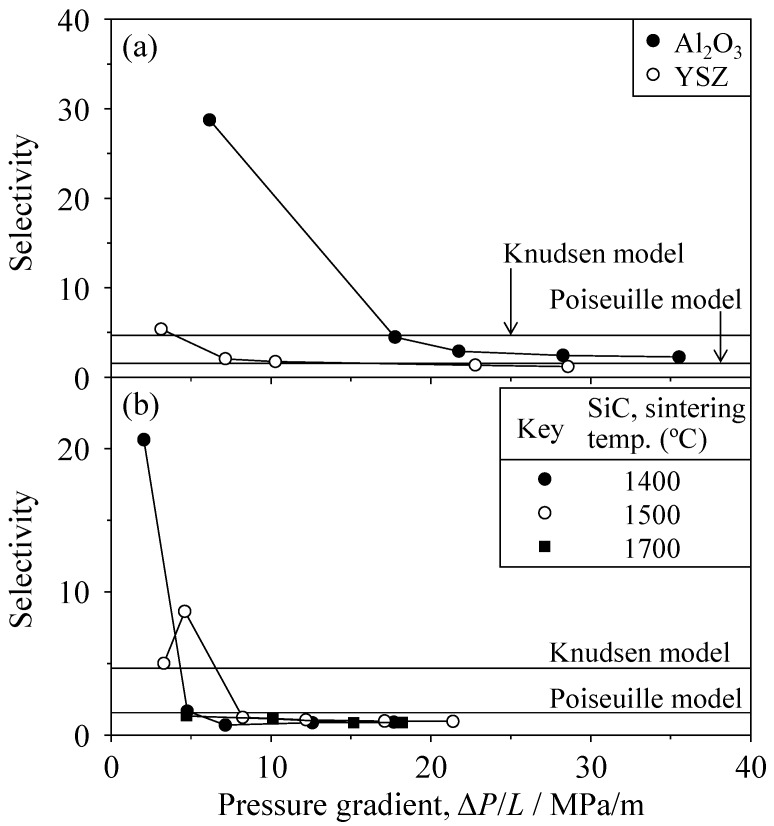
Selectivity (Equation (6)) of (**a**) Al_2_O_3_ and YSZ; and (**b**) SiC for the 50 vol % H_2_–50 vol % CO_2_ mixed gas.

The selectivity is defined generally by Equation (6) in the Introduction; the selectivity for 50% H_2_–50% CO_2_ mixed gas is included in Figure 16. The selectivity of all samples increased with decreasing pressure gradient. The selectivity of Al_2_O_3_ reached 28.8 at 6.16 MPa/m, which is significantly higher than the reported selectivity of 3.9 at Δ*P* = 0.1 MPa and 100 °C in the case of an amorphous silica membrane with a thickness of 30 nm [6].

### 3.4. Separation Model

In the present porous ceramics, the critical pressure gradient was measured in the single-gas H_2_ and CO_2_ gas flows (Figure 10). This property is affected by the attractive interaction between the pore walls and the polarized gas molecules. Furthermore, the critical pressure gradient is closely related to the tortuous pore structure in the porous ceramics, which interrupts the smooth transportation of gas molecules [17]. In the Knudsen and Poiseuille models, straight pores parallel to the direction of gas flow are assumed to transport the inlet gas molecules. The flux of the H_2_ gas of the mixed gas in the Knudsen model was modified by Equation (8) using the critical pressure gradient (∆*P*_c_/*L*) of single H_2_ gas flow:

(8) $$J(H_{2})=\frac{2\varepsilon r\bar{c}(H_{2})}{3RT}\frac{\Delta P(\mathrm{mix})x}{L}\left(1-\frac{\Delta P_{c}(H_{2})}{\Delta P(\mathrm{mix})}\right),$$

where Δ*P*(mix) is the pressure gradient of mixed gas, $\bar{c}(H_{2})$ is the velocity of H_2_ molecules, and *x* is the H_2_ gas fraction of the supplied mixed gas. This equation indicates that *J*(H_2_) becomes 0 mol/s m^2^ at *x* = 0 or ∆*P*(mix) = ∆*P*_c_(H_2_). Similarly, Equation (9) represents the flux of CO_2_ molecules of the mixed gas in the modified Knudsen model:

(9) $$J(CO_{2})=\frac{2\varepsilon r\bar{c}(CO_{2})}{3RT}\frac{\Delta P(\mathrm{mix})(1-x)}{L}\left(1-\frac{\Delta P_{c}(CO_{2})}{\Delta P(\mathrm{mix})}\right).$$

The H_2_ fraction is then expressed by Equation (10):

(10) $$F(H_{2})=\frac{J(H_{2})}{J(H_{2})+J(CO_{2})}=\frac{1}{1+\sqrt{\frac{M(H_{2})}{M(CO_{2})}}\frac{\left(\Delta P(\mathrm{mix})-\Delta P_{c}(CO_{2})\right)}{\left(\Delta P(\mathrm{mix})-\Delta P_{c}(H_{2})\right)}\frac{\left(1-x\right)}{x}}.$$

Equation (10) indicates that *F*(H_2_) approaches unity at *x* = 1 and Δ*P*(mix) = Δ*P*_c_(CO_2_). The flux values of H_2_ and CO_2_ molecules of the mixed gas in Poiseuille flow are modified by Equations (11) and (12), respectively:

(11) $$J(H_{2})=\frac{r^{2}\varepsilon \bar{P}(\mathrm{mix})}{8RT\eta (\mathrm{mix})}\frac{\Delta P(\mathrm{mix})x}{L}\left(1-\frac{\Delta P_{c}(H_{2})}{\Delta P(\mathrm{mix})}\right)$$

(12) $$J(CO_{2})=\frac{r^{2}\varepsilon \bar{P}(\mathrm{mix})}{8RT\eta (\mathrm{mix})}\frac{\Delta P(\mathrm{mix})(1-x)}{L}\left(1-\frac{\Delta P_{c}(CO_{2})}{\Delta P(\mathrm{mix})}\right).$$

In Equations (11) and (12), $\bar{P}(\mathrm{mix})$ is the average pressure between the inlet and outlet mixed gases, η(mix) is the viscosity of the mixed gas, and ∆*P*(mix)/*L* is the pressure gradient between the inlet and outlet mixed gases. The H_2_ fraction in the Poiseuille model is given by Equation (13):

(13) $$F(H_{2})=\frac{1}{1+\frac{\left(\Delta P(\mathrm{mix})-\Delta P_{c}(CO_{2})\right)}{\left(\Delta P(\mathrm{mix})-\Delta P_{c}(H_{2})\right)}\frac{\left(1-x\right)}{x}}.$$

Equation (13) approaches unity at *x* = 1 and ∆*P*(mix) = ∆*P*_c_(CO_2_) and is similar to Equation (10).

A specified pore provides different gas flow mechanisms for H_2_ and CO_2_ gases, as is evident in Figure 1 and Figure 9. H_2_ gas with λ/*r* > 1 and CO_2_ gas with λ/*r* < 1 in a specified pore are treated as Knudsen and Poiseuille flows, respectively. The H_2_ fraction of the mixed gas in this pore is derived from Equations (8) and (12) and is expressed by Equation (14):

(14) $$F(H_{2})=\frac{1}{1+\frac{3r\bar{P}(\mathrm{mix})}{16\eta (\mathrm{mix})\bar{c}(H_{2})}\frac{\left(\Delta P(\mathrm{mix})-\Delta P_{c}(CO_{2})\right)}{\left(\Delta P(\mathrm{mix})-\Delta P_{c}(H_{2})\right)}\frac{\left(1-x\right)}{x}}.$$

A commonality among Equations (10), (13), and (14) is the increase in the *F*(H_2_) value at a small ratio of *J*(CO_2_)/*J*(H_2_). This is discussed in the introduction and is seen in the calculated gas flux in Figure 6 or Figure 7. The theoretical H_2_ fraction increases in the order of Equation (13) (Poiseuille model) < Equation (10) (Knudsen model) < Equation (14) (Knudsen model for H_2_ gas, Poiseuille model for CO_2_ gas). That is, the specified pore giving Equation (14) exerts a kind of sieving effect for CO_2_ gas in the mixed gas. The previous discussion indicates that the following factors are key to increasing the H_2_ selectivity: (1) a large difference of ∆*P*_c_ for H_2_ and CO_2_ gases (∆*P*_c_(CO_2_) >> ∆*P*_c_(H_2_)), which is closely related to the tortuous pore structure, chemical composition of material, and Knudsen number (Figure 10); (2) a specified pore giving Knudsen flow for H_2_ gas and Poiseuille flow for CO_2_ gas (Figure 1 and Figure 9); and (3) attractive interactions between the pore walls and the specified gas molecules (CO_2_). In this paper, Factor (3) is not examined with sufficient experimental data.

**Figure 17:**
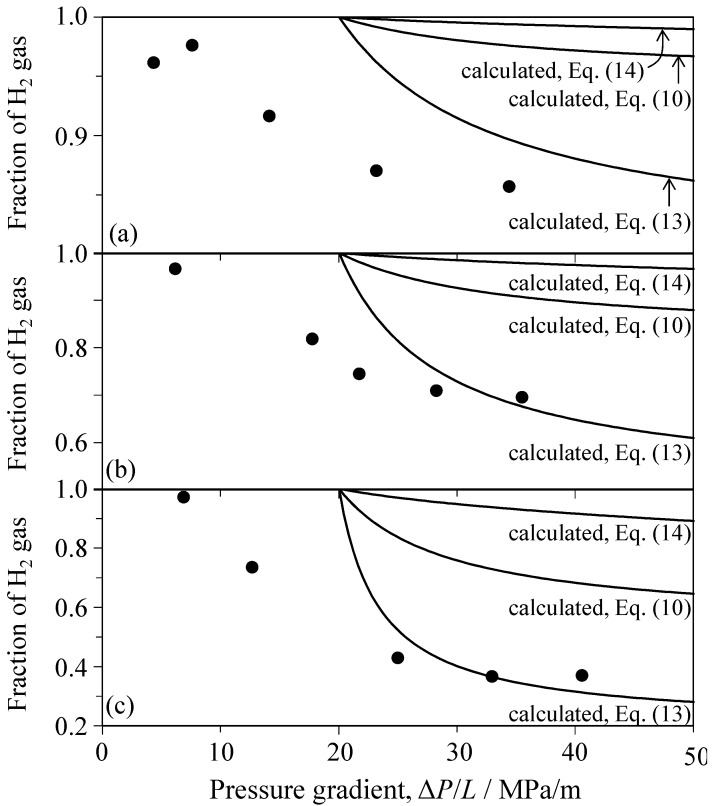
Comparison between measured and calculated H_2_ fractions in the porous Al_2_O_3_ ceramics for the supplied gas of (**a**) 80 vol % H_2_–20 vol % CO_2_; (**b**) 50 vol % H_2_–50 vol % CO_2_, and (**c**) 20 vol % H_2_–80 vol % CO_2_.

**Figure 18:**
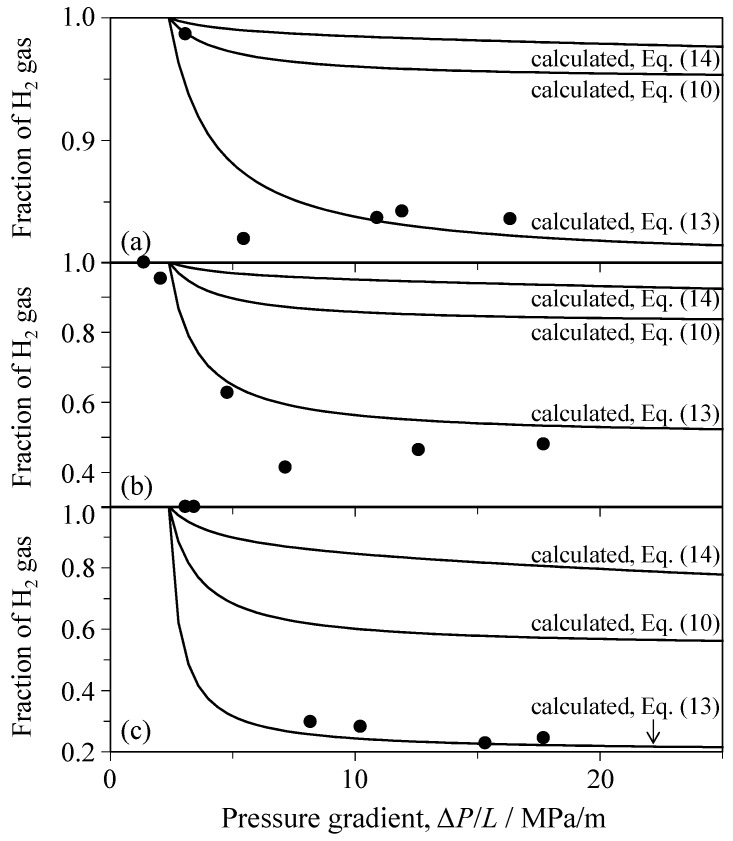
Comparison between measured and calculated H_2_ fractions in the porous SiC ceramics hot-pressed at 1400 °C for the supplied gas of (**a**) 80 vol % H_2_–20 vol % CO_2_; (**b**) 50 vol % H_2_–50 vol % CO_2_; and (**c**) 20 vol % H_2_–80 vol % CO_2_.

Figure 17 and Figure 18 show comparisons between measured and calculated H_2_ fractions in the Al_2_O_3_ porous ceramics and SiC porous ceramics, respectively. In the calculation of Equation (14), η(mix) is approximated by η(H_2_)*x* + η(CO_2_)(1 − *x*). As is evident in Figure 17 and Figure 18, the calculated *F*(H_2_) lines for Equations (10), (13) and (14) increase in the order Equation (13) < Equation (10) < Equation (14) with decreasing pressure gradient. The three calculated *F*(H_2_) lines of the mixed gas explain the general tendency of the experimental results. The discrepancy of ∆*P*/*L* values at *F*(H_2_) = 1 between the calculation and experiment in Figure 17 is related to the decrease of ∆*P*_c_(CO_2_) for the mixed gas as compared with single CO_2_ gas (Figure 11). The condition of ∆*P*_c_(CO_2_) ≈ ∆*P*_c_(H_2_) in Equations (10), (13) and (14) implies no dependence of *F*(H_2_) on ∆*P*(mix) and gives a constant *F*(H_2_) value that depends on the gas flow mechanism and the inlet gas composition (e.g., *F*(H_2_) = 0.5 in Equation (13), *F*(H_2_) = 0.824 in Equation (10), and *F*(H_2_) = 0.967 in Equation (14) for *x* = 0.5). The measured range of the H_2_ fraction for a constant *x* value in Figure 17 or Figure 18 was almost in agreement with the range calculated via Equation (13). This similarity suggests that (1) the mixed gas in the Al_2_O_3_ or SiC porous ceramics flows via the Poiseuille mechanism; (2) the flow mechanism of the mixed gas is controlled by CO_2_ gas with a small Knudsen number (Figure 9); and (3) the Knudsen flow of single H_2_ gas with a high Knudsen number is greatly influenced in the mixed gas by the coexisting CO_2_ molecules with a short molecular mean free path. Therefore, the molecular mean free path of H_2_ molecules is reduced by mixed CO_2_ molecules. That is, Equation (13) is able to explain the H_2_ fraction of the mixed gas under Poiseuille flow.

## 4. Reliability of Experiment

**Figure 19:**
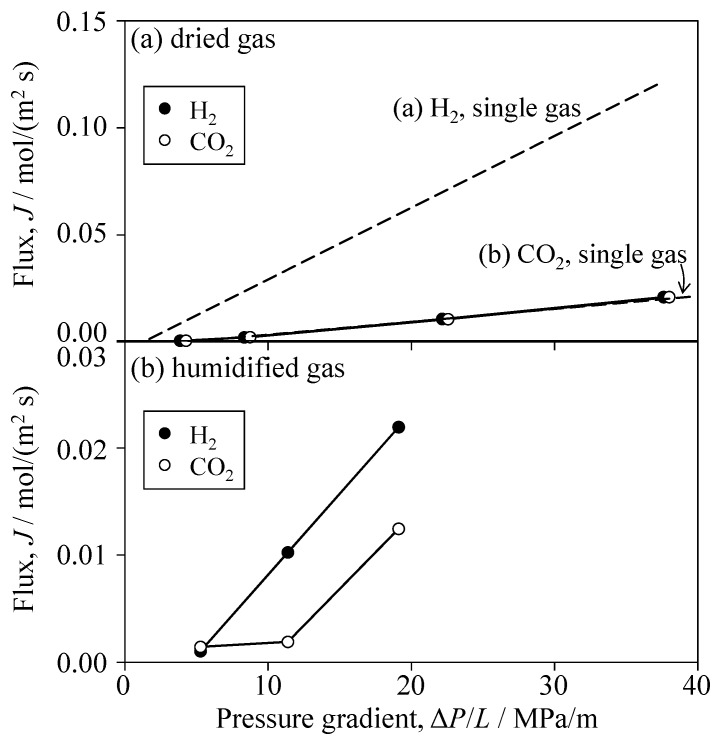
Flux of H_2_ and CO_2_ gases permeated through Al_2_O_3_ porous ceramics (60.9% relative density) for the 50 vol % H_2_–50 vol % CO_2_ mixed gas (**a**) with and (**b**) without 3 vol % H_2_O.

**Figure 20:**
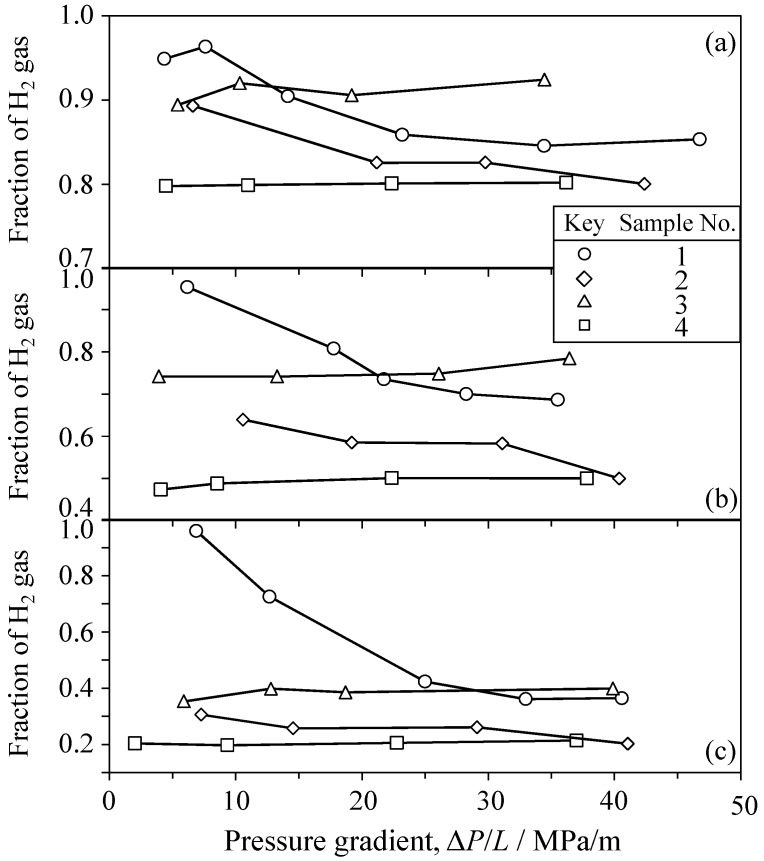
H_2_ fractions through the different four samples of Al_2_O_3_ sintered at 800 °C for the dried gas of (**a**) 80 vol % H_2_–20 vol % CO_2_; (**b**) 50 vol % H_2_–50 vol % CO_2_ and (**c**) 20 vol % H_2_–80 vol % CO_2_.

**Figure 21:**
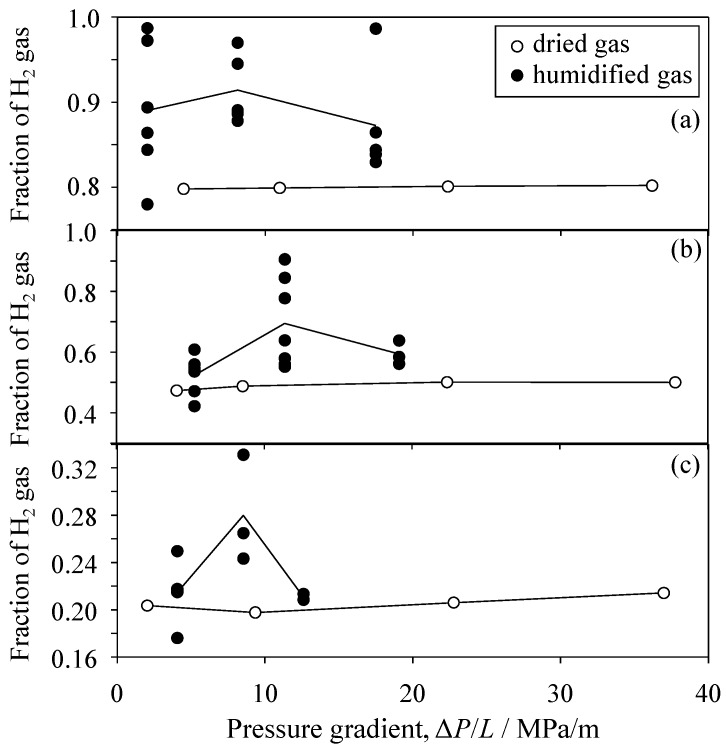
Influence of humidification of supplied gas of (**a**) 80 vol % H_2_–20 vol % CO_2_; (**b**) 50 vol % H_2_–50 vol % CO_2_ and (**c**) 20 vol % H_2_–80 vol % CO_2_ on the H_2_ fractions through the porous Al_2_O_3_ ceramics (60.9% relative density, sample No. 4 in Figure 20).

To demonstrate the reproducibility of the gas separation experiment, the experiments were repeated for another three Al_2_O_3_ samples with 59.3%–61.2% relative densities, which were sintered at 800 °C for 1 h. Figure 19 shows the typical flux values of the H_2_ and CO_2_ gases through the porous Al_2_O_3_ compact of 60.9% relative density (sample No. 4 in Figure 20) for the 50% H_2_–50% CO_2_ mixed inlet gas with and without moisture (3 vol % H_2_O). The humidification of the supplied gas was carried out by bubbling the mixed gas in distilled water at 25 °C. When the dried gas was supplied, the flux of H_2_ gas decreased by mixing as compared with the flux of single H_2_ gas. However, the CO_2_ gas flux of the mixed gas was not affected by coexisting H_2_ gas. These results were similar to the result shown in Figure 11. The critical pressure gradient of CO_2_ gas depended on the Al_2_O_3_ sample used. In 70% of the total 20 experiments using the different four Al_2_O_3_ samples, including the sample in Section 3, for several supplied gas compositions, the critical pressure gradient was observed for H_2_ and CO_2_ gases. Figure 20 shows the fraction of H_2_ gas permeated through the different four Al_2_O_3_ samples sintered at 800 °C. In two samples (Nos. 1 and 2), the H_2_ fraction increased with a decrease in the pressure gradient. The other two samples (Nos. 3 and 4) exhibited no dependence of H_2_ fraction on pressure gradient, but the H_2_ fraction of sample No. 3 was 10%–25% higher than that of the supplied gas. Therefore, in 75% of the experiments with the different four samples and several supplied gas compositions, the fraction of permeated H_2_ gas increased up to 16%–76% from the H_2_ fraction of the supplied gas. The influence of moisture in the porous compact or in the H_2_–CO_2_ mixed gas was investigated using sample No. 4, Al_2_O_3_ (60.9% relative density) in Figure 19 and Figure 21. When the humidified H_2_–CO_2_ mixed gas was supplied to sample No. 4, Al_2_O_3_, the dependence of the CO_2_ gas flux on the pressure gradient (Figure 19b) was similar to the trend described in Section 3.3 (Figure 11). That is, the CO_2_ gas flux decreased rapidly with decreasing pressure gradient. In the dried gas condition, the H_2_ fraction was almost the same as that of supplied gas in the pressure gradient range from 2.0 to 37.8 MPa/m (Figure 21). In the humidified condition, the fraction of H_2_ gas, which was measured 2–6 times at each pressure gradient, was significantly scattered but the H_2_ fraction was apparently enhanced as compared to the dried gas condition. The maximum H_2_ fraction was 0.331, 0.905, and 0.986 when the supplied gas contained 20%, 50%, and 80% H_2_, respectively. Therefore, the moisture in the experimental condition adsorbs easily on the present porous ceramics, as described in the experimental section, and affects the selectivity of the supplied H_2_–CO_2_ mixed gas. After the experiment with the humidified gas in Figure 21, a slight weight gain of 0.034% was measured. That is, the scattered data of the gas separation in Figure 21 are caused by the structure of porous ceramics (tortuosity and size distribution of pores, and porosity) and interaction between gas molecules and pore wall (i.e., ceramics particles), which is affected by the moisture adsorbed on the porous ceramics or included in the supplied gas.

## 5. Conclusions

Porous ceramics of α-alumina, yttria-stabilized zirconia (YSZ), and silicon carbide were fabricated, and their permeability toward hydrogen, carbon dioxide, and hydrogen–carbon dioxide mixtures was investigated. The open porosity and median pore size of the fabricated porous ceramics were 36.2% and 45.3 nm for Al_2_O_3_, 40.5% and 115.8 nm for YSZ, and 17.9%–36.1% and 111.9–164.0 nm for SiC samples hot-pressed at 1400–1700 °C, respectively. The permeation of single H_2_ or CO_2_ gas occurred above a critical pressure gradient and increased linearly with an increasing pressure gradient. A higher flux and a lower critical pressure gradient were measured for H_2_ gas than for CO_2_ gas. In the Al_2_O_3_ and YSZ porous ceramics, the permeability coefficients of both H_2_ and CO_2_ gases were similar to those predicted by the Knudsen model. In the SiC porous ceramics, the measured permeability coefficient of H_2_ gas was explained by the Knudsen model, and the permeability coefficient of CO_2_ gas was within the range based on values calculated by the Knudsen and Poiseuille models. The aforementioned results are explained by the Knudsen number (λ/*r* ratio, λ: molecular mean free path of gas molecules, *r*: pore radius). When the Knudsen number is larger than unity, Knudsen flow becomes a dominant gas transportation process. In the nanometer-sized tortuous pore structure, the composition of material and the Knudsen number (λ/*r* ratio) affect the critical pressure gradient of a single gas of H_2_ or CO_2_. The critical pressure gradient increases more with decreasing Knudsen number in the case of Al_2_O_3_ than in the case of SiC.

In the permeation of the mixed gas of 50 vol % H_2_–50 vol % CO_2_, the H_2_ flux value in Al_2_O_3_ or YSZ became smaller than that of single H_2_ gas and approached that of single CO_2_ gas. By contrast, the CO_2_ flux in the mixed gas through SiC ceramics became larger than that of single CO_2_ gas and approached the flux of single H_2_ gas. The fraction of H_2_ gas through the Al_2_O_3_ and YSZ compacts became higher than the mixing ratio of the supplied gas and increased with decreasing pressure gradient, reaching 0.966 at 6.16 MPa/m in Al_2_O_3_ and 0.844 at 3.13 MPa/m in YSZ. These values of H_2_ fraction exceeded the fraction calculated using the Knudsen equation (0.824). In porous SiC, the H_2_ fraction increased at a lower pressure gradient (<10 MPa/m) and reached 0.999 at 1.4 MPa/m of pressure gradient. A similar trend of the dependence of the H_2_ fraction on the pressure gradient was also observed for the mixed gases with different inlet gas compositions of 20 vol % H_2_–80 vol % CO_2_ and 80 vol % H_2_–20 vol % CO_2_. When 80% H_2_–20% CO_2_ mixed gas was permeated, the H_2_ fraction was 0.976 at 7.61 MPa/m in Al_2_O_3_, 0.936 at 3.13 MPa/m in YSZ, and 0.987 at 3.06 MPa/m in SiC, respectively. In the case of 20% H_2_–80% CO_2_ mixed gas, the H_2_ fraction was 0.972 at 6.88 MPa/m in Al_2_O_3_, 0.504 at 8.04 MPa/m in YSZ and 0.999 at 3.40 MPa/m in SiC, respectively. The aforementioned high fraction of H_2_ gas was closely related to the difference in the critical pressure gradient values for H_2_ and CO_2_ single gases, the inlet mixed gas composition, and the gas flow mechanism of the mixed gas. A gas flow of CO_2_ molecules with a small Knudsen number and the collision between H_2_ and CO_2_ molecules control the gas flow mechanism (Poiseuille flow) of the mixed gas. The calculated H_2_ fraction derived from the modified Poiseuille equations of H_2_ and CO_2_ gases explained well the measured H_2_ fraction as a function of pressure gradient of the mixed gas in Al_2_O_3_ and SiC porous ceramics. In the present experimental conditions, moisture in atmosphere adsorbed easily on the porous ceramics and affected the critical pressure gradient, leading to the increased selectivity of H_2_ gas.

## Figures and Tables

**Table 1:** Summary of permeability coefficients in Figure 9.

Gas	Sample	Knudsen Number	Permeability Coefficient (10^−9^ mol·m/(m^2^·s·Pa))		
			Measured	Calculated	
				Knudsen	Poiseuille
H_2_	Al_2_O_3_	3.62–4.57	3.71–3.97	3.96	0.13–0.16
	YSZ	1.65–1.91	13.6–13.7	11.3	0.87–1.01
	SiC, 1400 °C	1.80–2.11	7.26–15.0	9.75	0.68–0.80
	SiC, 1500 °C	1.78–2.03	2.12–3.75	7.72	0.56–0.64
	SiC, 1700 °C	1.22–1.44	2.38–15.4	7.08	0.73–0.86
CO_2_	Al_2_O_3_	1.30–1.41	1.35–1.48	0.85	0.09–0.10
	YSZ	0.59–0.62	2.88–3.47	2.42	0.06
	SiC, 1400 °C	0.56–0.72	0.18–0.48	2.09	0.42–0.54
	SiC, 1500 °C	0.50–0.64	0.43–1.82	1.65	0.38–0.49
	SiC, 1700 °C	0.41–0.49	0.87–2.82	1.52	0.45–0.55
